# Reduction of benzylic alcohols and α-hydroxycarbonyl compounds by hydriodic acid in a biphasic reaction medium

**DOI:** 10.3762/bjoc.8.36

**Published:** 2012-03-02

**Authors:** Michael Dobmeier, Josef M Herrmann, Dieter Lenoir, Burkhard König

**Affiliations:** 1 Institute of Organic Chemistry, University of Regensburg, D-93040 Regensburg, Germany, Fax: +499419431717; 2 Helmholtz Zentrum München, Ingolstädter Landstraße 1, D-85764 Neuherberg, Germany

**Keywords:** alcohols, catalysis, iodine, phosphorous reduction

## Abstract

The synthetic protocol for the reduction of alcohols to hydrocarbons by using hydriodic acid, first described by Kiliani more than 140 years ago, was improved to be more applicable to organic synthesis. Instead of a strongly acidic, aqueous solution, a biphasic toluene–water reaction medium was used, which allowed the conversion of primary, secondary and tertiary benzylic alcohols, in good yields and short reaction times, into the corresponding hydrocarbons. Red phosphorous was used as the stoichiometric reducing agent. Keto, ester, amide or ether groups are tolerated, and catalytic amounts of hydriodic acid (0.2 equiv) in the presence of 0.6 equiv phosphorous are sufficient to achieve conversion.

## Introduction

The reduction of hydroxy groups is a typical and important step in the synthesis of complex natural products or drugs [1–4]. Functional-group tolerance during this reduction step is essential since various other groups are usually present. A number of synthetic procedures have been developed, which allow selective reduction, but only a few one-step transformations are known, which use either titanium(III) [5–8] or different metal complexes [9–13]. Most procedures require a sequence of steps, e.g., the conversion of hydroxy groups into a chloride or bromide substituent and subsequent catalytic reduction with H_2_/Pt or the conversion into a tosylate and reduction with LiAlH_4_. The most commonly applied method is the Barton–McCombie reaction [14], due to its versatility and its very high functional-group tolerance [15–18]. Although very general, the reaction has some drawbacks: The involved organotin hydrides are costly, highly toxic [19–21] and often difficult to separate from the reaction products. Furthermore, secondary alcohols give best results, while others may react less efficiently.

We have reinvestigated the long-known reduction of benzylic alcohols and α-hydroxycarbonyl compounds by hydriodic acid [22–32]. The method has been reported for a variety of alcohols, but typically proceeds in aqueous solution and requires an excess of HI or strong mineral acids such as phosphoric or sulfuric acid [33–35]. We describe a biphasic reaction medium consisting of toluene and aqueous hydriodic acid. The phase separation allows milder reaction conditions compared to the classic Kiliani protocol and is more applicable to organic synthesis.

## Results and Discussion

Initial investigations focused on simple benzylic alcohols (Table 1, entries 1–3), which were converted in high to quantitative yields into the corresponding alkanes. Carbonyl groups or amides in a benzylic position (Table 1, entries 4 and 6) and aromatic hydroxy groups (Table 2, entry 7) or aromatic ethers (Table 1, entry 5) were not affected. Moreover, heterocycles such as thiophene (Table 1, entry 7) were stable under these conditions whereas furans (Table 1, entry 8) were decomposed due to ring opening. Benzylic alcohols were converted in good to high yields to alkanes with increasing reactivity in the order primary (2 h) < secondary (0.5–1 h) < tertiary alcohol (15–30 min); carbonyl groups and ethers were tolerated. Diethyl tartrate was converted into diethyl succinate under the reaction conditions given (Table 1, entry 12), but some of the material was lost due to ester hydrolysis.

**Table 1:** Reduction of benzylic alcohols to the corresponding alkanes.

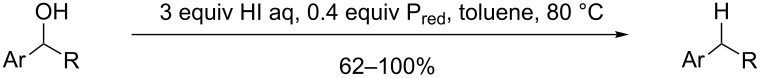				

Entry	Alcohol	Product^a^	Time [h]	Yield [%]

1	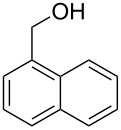	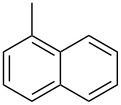	2	70^b^
2	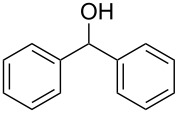	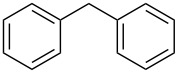	0.5	96
3	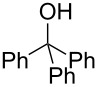	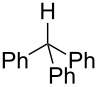	0.25	100
4	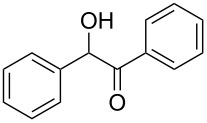	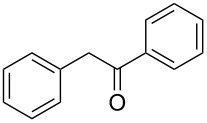	1	80
5	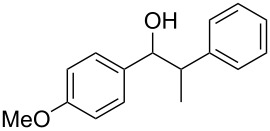	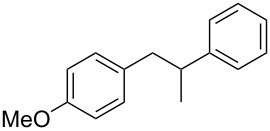	0.5	92
6	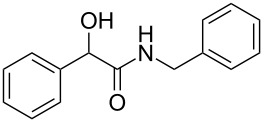	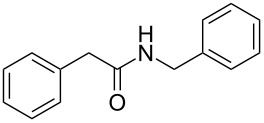	1	82
7	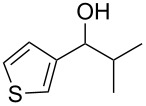	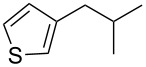	0.5	62^c^
8	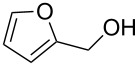	decomposition	1	–
9	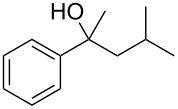	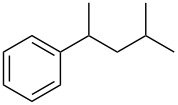	0.25	74^c^
10	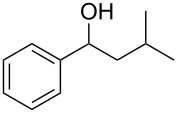	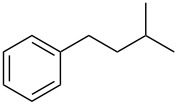	0.5	49^c^
11	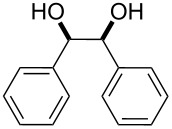	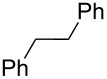	0.5	78
12	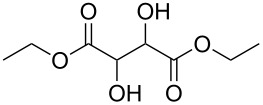	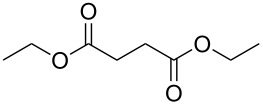	1.5	65

^a^All products are known compounds described in the literature. The identities have been proven by proton NMR and mass analysis, which match the literature data. ^b^The corresponding iodo compound was identified as a byproduct. ^c^The corresponding elimination product was obtained as a byproduct.

**Table 2:** Alcohols showing incomplete or unselective reaction with hydriodic acid and red phosphorous (3.0 equiv HI, 0.4 equiv P_red_).

Entry	Alcohol	Product	Time [h]	Yield [%]

1	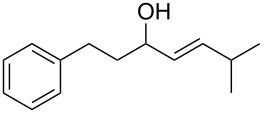	mixture of several products	1	–
2	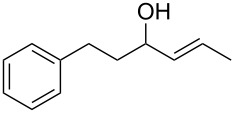	mixture of several products	1	–
3	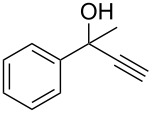	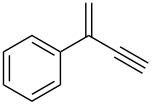	1	traces
4	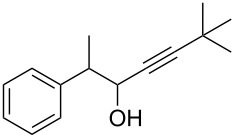	decomposition	1	–
5	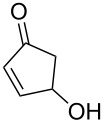	decomposition	1	–
6	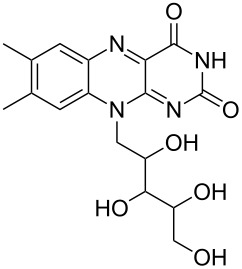	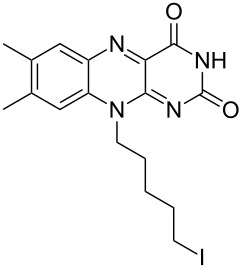	2	21
7	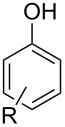	no conversion	1	–

Allylic alcohols are completely consumed, but the corresponding alkenes could not be isolated as pure products (Table 2). Mixtures of eliminiation and deoxygenation products, in some cases also rearangement of the deoxygenated product into the the more highly substitued, thermodynamically more stable alkene occurred. Propargylic alcohols (Table 2, entry 3 and 4) showed elimination or decomposed. In the case of flavin (Table 2, entry 6), three hydroxy groups were reduced and one was converted into an iodo substituent.

Alcohols other than those that were benzylic or α to carbonyl groups were not converted into the corresponding alkanes, and the reaction stopped at the iodoalkanes (Table 3). The reactivity follows the order of primary < secondary < tertiary alcohols, as expected for an S_N_1 reaction. The reduction potential of the nonbenzylic iodoalkanes is not sufficient for reduction by hydriodic acid.

**Table 3:** Alcohols yielding alkyl iodides with hydriodic acid and red phosphorous.^a^

Entry	Alcohol	Product	Time [h]	Yield [%]

1	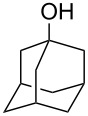	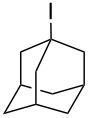	8	98
2	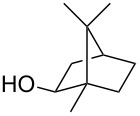	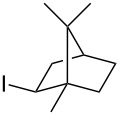	8	83^b^
3	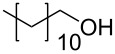	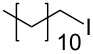	20	81^c^

^a^3 equiv HI, 0.4 equiv P_red_. ^b^Single isomer. ^c^Products were analyzed by gas chromatography; chlorobenzene was used as an internal standard.

**Scheme 1:**
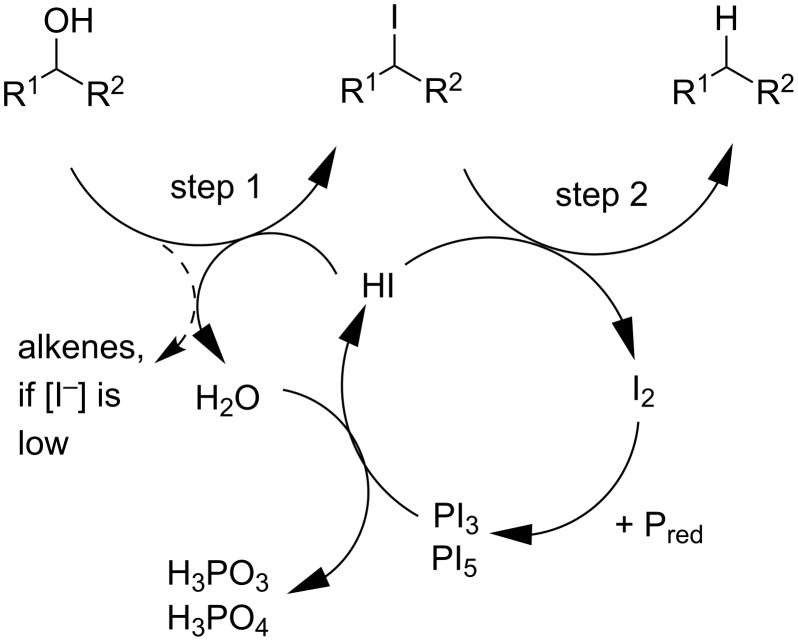
Mechanism of the alcohol reduction and recycling of iodine.

The mechanism of reduction by hydriodic acid consists of two steps (Scheme 1): The nucleophilic substitution of the hydroxy group by iodide and the subsequent reduction of the alkyl iodide by hydriodic acid. The mechanistic details of the redox comproportionation of alkyl iodides and H–I have been strongly debated in the literature [36–38]. However, the required benzylic or α-carbonyl position for the redox comproportionation indicates an intermediate with mesomeric stabilization due to the adjacent π-system. In a trapping experiment, using HI without phosphorous, diphenylcarbinol as the substrate and TEMPO as a trapping agent for radical intermediates, the TEMPO adduct of diphenylcarbinol was detected by mass analysis. This indicates a radical mechanism of the redox comproportionation. We suggest a stepwise reduction by single electron transfer (SET) accompanied by the oxidation of I^−^ to I_2_. The iodine, generated in the second step, is recycled by reduction with red phosphorous, regenerating hydriodic acid. Admittedly, the above-mentioned TEMPO adduct could also be generated by nucleophilic substitution of the alkyl iodide with reduced TEMPO. At least this would be another proof for the first reaction step. According to the redox equations of the reaction between iodine and red phosphorous, each mole of red phosphorous is able to reduce at least 1.5 mol of iodine. Catalytic amounts of hydriodic acid are therefore sufficient [28] for the reduction of the hydroxy group (Table 4), when excess red phosphorous is added as a terminal reducing agent. However, depending on the amount of added hydriodic acid, the elimination of water may occur as an alternative reaction pathway. A low concentration of HI favors the elimination of water, while higher HI concentrations lead to substitution and reduction products.

**Table 4:** Reduction of alcohols with catalytic amounts of hydriodic acid.

Entry	Alcohol	Product	Time [h]	Yield [%]

1	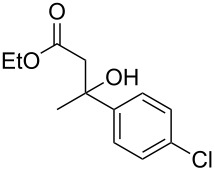	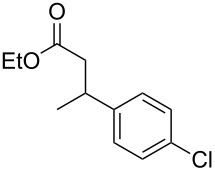	0.5	82^a^
2	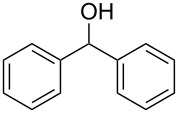	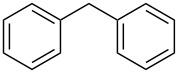	0.5	92^b^
3	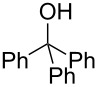	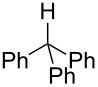	0.25	98^b^
4	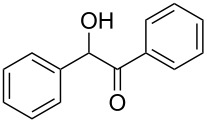	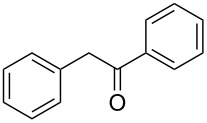	0.5	74^b^

^a^0.6 equiv HI, 0.4 equiv P_red_. ^b^0.1 equiv HI, 0.7 equiv P_red_.

## Conclusion

Toluene and aqueous hydriodic acid are a suitable biphasic reaction mixture for the reduction of a range of benzylic alcohols. The two-phase system makes the Kiliani protocol more easily applicable to organic synthesis, as organic substrates and products dissolve in the organic phase and are separated from the mineral acids. The procedure allows the use of catalytic amounts of hydriodic acid and red phosphorous as the terminal reductant. In the case of alcohols having no activation by adjacent benzylic or carbonyl groups the reaction stops at the corresponding alkyl iodide. A quantitative mass-efficiency analysis [39] of the reaction in comparison to tosylation/LAH, Ti(III)-mediated and Barton–McCombie reduction revealed a better atom economy and mass efficiency.

## Experimental

Representative experimental procedure: The alcohol (1 mmol, 1 equiv) is dissolved in 4 mL of toluene. Red phosphorus (0.4 mmol), followed by concentrated hydriodic acid (57% w/w; 3.0 mmol, 3 equiv) is added and the reaction mixture is heated to 80 °C for the stated time, allowed to cool to rt and quenched with Na_2_S_2_O_3_ (10 mL; 10% w/w) solution. The aqueous phase is extracted with dichloromethane (3 × 10 mL), the combined organic phases are dried over MgSO_4_ and filtered, and the solvent is removed. The crude product is purified by chromatography and spectroscopically characterized.

For catalytic reactions of 1 mmol of the respective alcohol the following amounts of hydriodic acid and P_red_ were used: (a) 0.6 mmol HI/0.4 mmol P_red_, (b) 0.1 mmol HI/0.7 mmol P_red_.

**(*E*)-6-Methyl-1-phenylhept-4-en-3-ol** (Table 2, entry 1): The reaction was carried out under dry nitrogen atmosphere by using standard Schlenk techniques. To a slurry of Mg powder (0.67 g, 28 mmol) in dry THF (4 mL), 2 mL of a solution of 2-phenyl-1-bromethane (3.0 mL, 28 mmol) in dry THF (10 mL) was added. The Grignard reaction was initiated by the addition of iodine followed by sonication for several minutes. When the exothermic reaction started the rest of the 2-phenyl-1-bromethane solution was added through a septum by syringe over 15 min. After the addition, the reaction solution was heated under reflux for 1 h to complete the reaction. The reaction solution was allowed to cool to rt before 4-methyl-2-pentenal (2.3 mL, 20 mmol) was added dropwise. To complete the reaction the solution was again heated under reflux for 1 h. The reaction was quenched by the addition of HCl (2 M, 25 mL). The aqueous phase was extracted with diethyl ether (3 × 15 mL). The combined organic phases were washed with saturated NaHCO_3_ (15 mL) and H_2_O (2 × 10 mL), and dried with MgSO_4_. The solvent was removed with a rotary evaporator. The crude product was purified by flash chromatography (petroleum ether/ethyl acetate 4:1, *R*_f_ 0.32; staining with vanillin solution gave a blue spot). (*E*)-6-Methyl-1-phenylhept-4-en-3-ol was isolated as a yellow oil in 74% yield (3.05 g, 14.9 mmol). ^1^H NMR (300 MHz, CDCl_3_) δ 7.33–7.14 (m, 5H), 5.63 (ddd, *J* = 15.5, 6.4, 0.7 Hz, 1H), 5.44 (ddd, *J* = 15.5, 7.0, 1.2 Hz, 1H), 4.13–4.01 (m, 1H), 2.79–2.59 (m, 2H), 2.39–2.21 (m, 1H), 1.97–1.72 (m, 2H), 1.58 (d, *J* = 2.7 Hz, 0.3H), 1.46 (d, *J* = 1.8 Hz, 1H), 1.00 (d, *J* = 6.8 Hz, 6H); ^13^C NMR (75 MHz, CDCl_3_) δ 139.6, 129.7, 128.5, 128.4, 125.8, 72.6, 38.8, 31.8, 30.7, 22.4, 21.3; EIMS *m*/*z* (%): 91.1 (100) [C_7_H_7_]^+^, 161.1 (81) [M − C_3_H_7_]^+^, 186.1 (5) [M − H_2_O]^+^, 204.2 [M]^+∙^; HRMS (*m*/*z*): [M]^+^ calcd for C_14_H_20_O, 204.1514; found, 204.1511.

***(E*)-1-Phenylhex-4-en-3-ol** (Table 2, entry 2): The reaction was carried out under a dry nitrogen atmosphere by using standard Schlenk techniques. A solution (1 mL) of 2-phenyl-1-bromethane (1.35 mL, 10.0 mmol) in dry THF (10 mL) was added to Mg powder (0.25 g, 10 mmol). The Grignard reaction was initiated by the addition of iodine followed by sonication for several min. When the exothermic reaction started the rest of the 2-phenyl-1-bromethane solution was added through a septum by syringe over 15 min. After the addition, the reaction solution was heated under reflux for 1 h to complete the reaction. The reaction solution was allowed to cool to rt before crotonaldehyde (0.74 mL, 9.0 mmol) was added dropwise. To complete the reaction the solution was again heated under reflux for 2.5 h. The reaction was quenched by the addition of HCl (2 M, 10 mL). The aqueous phase was extracted with diethyl ether (2 × 15 mL). The combined organic phases were washed with saturated NaHCO_3_ (5 mL), H_2_O (2 × 5 mL) and dried with MgSO_4_. The solvent was removed with a rotary evaporator. (*E*)-1-Phenylhex-4-en-3-ol was obtained in 96% yield (1.53 g, 8.69 mmol) in analytical purity. Analytical data were identical with the literature [40]. ^1^H NMR (300 MHz, CDCl_3_) δ 7.34–7.06 (m, 5H), 5.63 (dq, *J* = 15.3, 6.2 Hz, 1H), 5.48 (ddd, *J* = 15.3, 7.0, 1.4 Hz, 1H), 4.02 (q, *J* = 6.7 Hz, 1H), 2.73–2.56 (m, 2H), 1.67 (dd, *J* = 6.3, 0.7 Hz, 3H), 1.52 (s, 0.3H), 1.40 (s, 0.7H); EIMS *m*/*z* (%): 71.1 (100) [C_4_H_7_O]^+^, 91.1 (67) [C_7_H_7_]^+^, 105.1 (19) [M − C_4_H_7_O]^+^, 176.1 (50) [M]^+^.

**1-(4-Methoxyphenyl)-2-phenylpropan-1-ol (**Table 1, entry 5): The reaction was carried out under a dry nitrogen atmosphere by using standard Schlenk techniques. 1 mL of a solution of 4-bromo-1-methoxybenzene (0.62 mL, 5.0 mmol) in dry THF (10 mL) was added to Mg powder (0.12 g, 5.0 mmol). The Grignard reaction was initiated by the addition of iodine followed by sonication for several min. When the exothermic reaction started the rest of the 4-bromo-1-methoxybenzene solution was added through a septum by syringe over 15 min. After the addition, the reaction solution was heated under reflux for 1 h to complete the reaction. The reaction solution was allowed to cool to rt before 2-phenylpropionaldehyde (0.60 mL, 4.5 mmol) was added dropwise. To complete the reaction the solution was again heated under reflux for 2 h. The reaction was quenched by the addition of HCl (2 M, 5 mL). The aqueous phase was extracted with diethyl ether (2 × 5 mL). The combined organic phases were washed with saturated NaHCO_3_ (3 mL), H_2_O (2 × 2.5 mL) and dried with MgSO_4_. The solvent was removed with a rotary evaporator. The crude product was purified by flash chromatography (petroleum ether/ethyl acetate 4:1, *R*_f_ 0.3; staining with vanillin solution gave a blue spot). 1-(4-Methoxyphenyl)-2-phenylpropan-1-ol was isolated as a yellow oil in 57% yield (0.62 g, 2.6 mmol). Analytical data are identical with literature [41]. ^1^H NMR (300 MHz, CDCl_3_) δ 7.45–7.05 (m, 7H), 6.85–6.74 (m, 2H), 4.76 (d, *J* = 6.1 Hz, 1H), 3.78 (s, 3H), 3.09 (p, *J* = 6.9 Hz, 1H), 1.34 (d, *J* = 7.0 Hz, 3H); EIMS *m*/*z* (%): 137.1 (53) [M − C_8_H_9_]^+^, 224.1 (2) [M − H_2_O]^+^, 242.1 (1) [M]^+∙^.

**6,6-Dimethyl-2-phenylhept-4-yn-3-ol** (Table 2, entry 4): The reaction was carried out under a dry nitrogen atmosphere by using standard Schlenk techniques. The solution of 3,3-dimethyl-1-butyne (0.62 mL, 5 mmol) in dry THF (10 mL) was cooled to −78 °C. *n*-BuLi (1.6 M in hexane, 3.5 mL, 5.6 mmol) was added dropwise through a septum by syringe. The reaction mixture was allowed to warm to rt before the solution of 2-propionaldehyde (0.68 mL, 5 mmol) in dry THF (5 mL) was added dropwise through a septum by syringe. This solution was stirred for 4.5 h. The reaction was stopped by the addition of H_2_O (10 mL). The aqueous phase was extracted with diethyl ether (3 × 15 mL), and the combined organic layers were dried with MgSO_4_. The solvent was removed with a rotary evaporator. The crude product was purified by flash chromatography (petroleum ether/ethyl acetate 4:1, *R*_f_ 0.42; staining with vanillin solution gave a blue spot). 6,6-dimethyl-2-phenylhept-4-yn-3-ol was isolated as a colorless oil in 46% yield (0.50 g, 2.3 mmol). ^1^H NMR (300 MHz, CDCl_3_) δ 7.40–7.19 (m, 5H), 4.44 (dd, *J* = 7.4, 5.4 Hz, 1H), 3.03 (dd, *J* = 7.1, 5.4 Hz, 1H), 1.67 (d, *J* = 5.4 Hz, 1H), 1.64 (d, *J* = 7.4 Hz, 1H), 1.39 (d, *J* = 7.1 Hz, 3H), 1.17 (s, 9H); ^13^C NMR (75 MHz, CDCl_3_) δ 141.9, 128.8, 128.2, 127.0, 95.5, 78.1, 67.8, 67.5, 55.0, 46.1, 31.0, 30.0, 16.3; EIMS *m*/*z* (%): 57.1 (36) [C_4_H_9_]^+^, 99.1 (100), 105.1 (20) [C_8_H_10_]^+^, 216.2 (7) [M]^+∙^.

## Supporting Information

File 1 Spectroscopic data for the synthesis of some alcohols. Quantitative mass efficiency analysis of four alternative alcohol reduction reactions.
