# Supplementary material for: Reduction of benzylic alcohols and α-hydroxycarbonyl compounds by hydriodic acid in a biphasic reaction medium
Source: Beilstein J Org Chem. 2012 Mar 2;8:330–6. doi: 10.3762/bjoc.8.36 (PMC3302097; doi:10.3762/bjoc.8.36)
Supplement: File 1 — Spectroscopic data for the synthesis of some alcohols. Quantitative mass efficiency analysis of four alternative alcohol reduction reactions. [file Beilstein_J_Org_Chem-08-330-s001.pdf]

Supporting Information

for

Reduction of benzylic alcohols and α-hydroxy carbonyl compounds by hydriodic acid in a biphasic reaction medium

Michael Dobmeier[1], Josef M. Herrmann[1], Dieter Lenoir[2] and Burkhard König*[1]

Address: [1]Institute of Organic Chemistry, University of Regensburg, D-93040 Regensburg, Germany, Fax: +499419431717 and [2]Helmholtz Zentrum München, Ingolstädter Landstraße 1, D-85764 Neuherberg, Germany

E-mail: Burkhard Koenig - Burkhard.koenig@chemie.uni-regensburg.de

*Corresponding author

Spectroscopic data for the synthesis of some alcohols. Quantitative mass efficiency analysis of four alternative alcohol reduction reactions.

Table of contents

1. General methods S2
2. Copies of NMR spectra S3
3. Mass efficiency analysis of alternative alcohol deoxygenation methods S4

1. General methods

All reagents and solvents used were of analytical grade, purchased from commercial sources and used without further purification. Unless stated otherwise, purification and drying of the solvents used was performed according to accepted general procedures. All reactions were performed under an inert atmosphere of N_2 by using standard Schlenk techniques, if not otherwise stated. TLC analyses were performed on silica-gel-coated alumina plates (F^{254} silica gel, layer thickness 0.2 mm). Visualization was achieved by UV light at 254 nm/366 nm or through staining with ninhydrin dissolved in EtOH. For preparative column chromatography, silica gels (70–230 mesh and 230–400 mesh) were used. For chromatography commercially available solvents of standard quality were used without further purification.

2. Copies of ^{1}H and ^{13}C NMR spectra

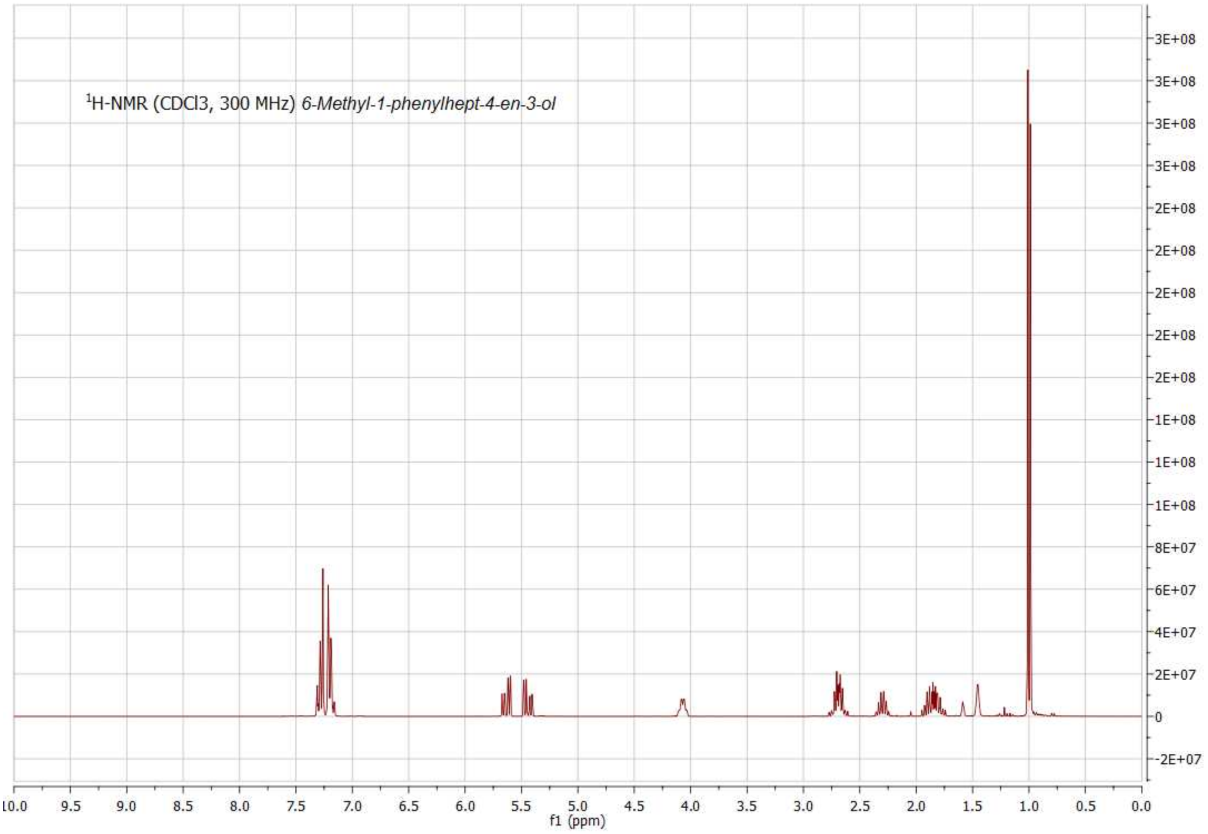

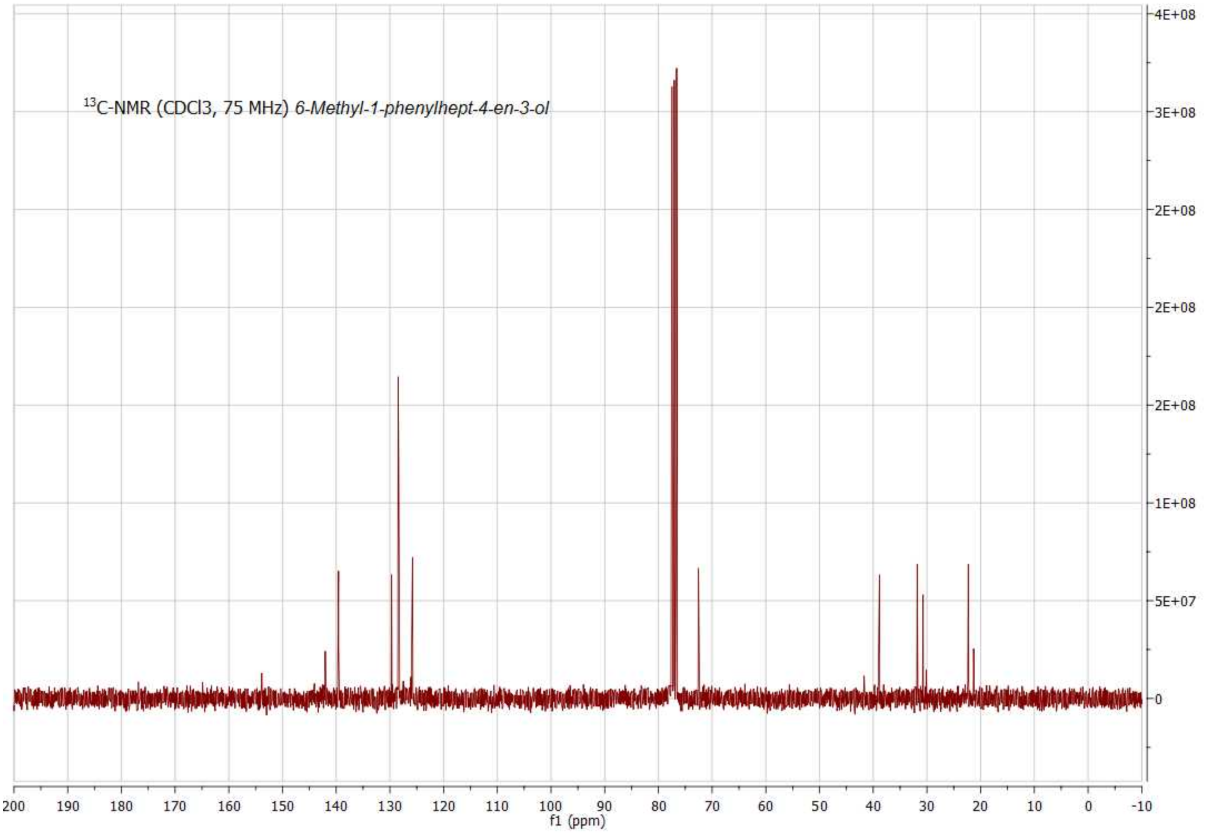

3. Mass efficiency analysis of alternative alcohol deoxygenation methods

To allow for a more quantitative comparison of the reduction method described herein with regard to environmental aspects and efficiency we determined the E factor of four alternative reactions for the synthetic step (Scheme S1 to Scheme S4).

$$E = \frac{\text{Waste [kg]}}{\text{Product [kg]}}$$

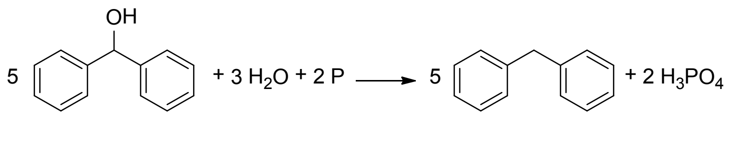

Scheme S1: Reduction of an alcohol by phosphorous (analogously to Table 1, entry 2).

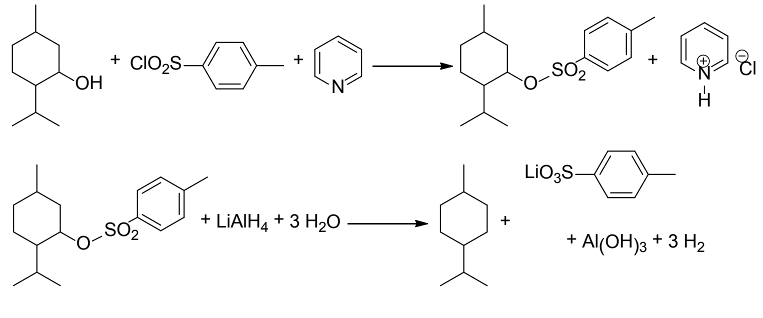

Scheme S2: Lithium aluminium hydride as reducing agent for alcohols [1,2].

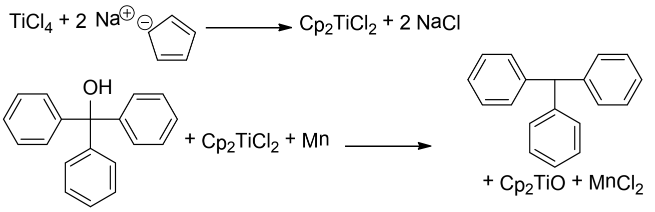

Scheme S3: Ti(III)-mediated reduction of alcohols [3].

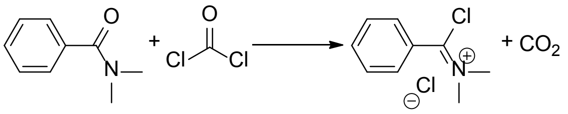

a)

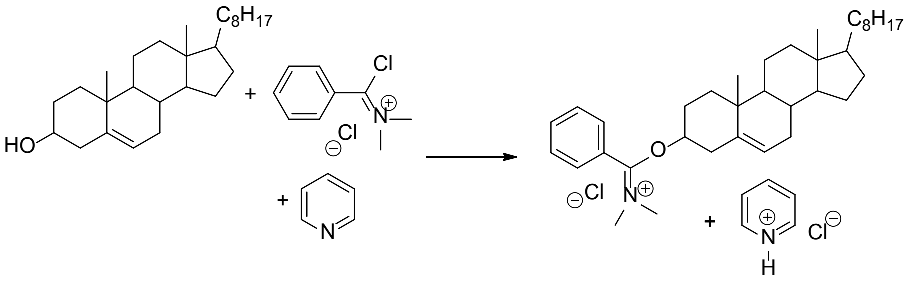

b)

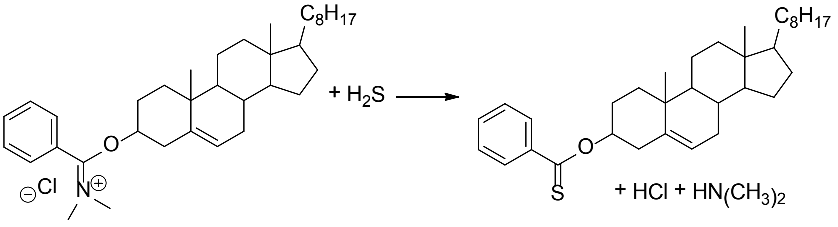

c)

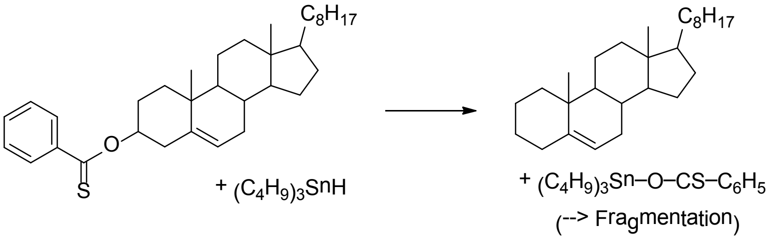

d)

Scheme S4: Tributyl stannane as reducing agent in a synthesis sequence (a–d) resulting in cholest-5-ene [4].

The protocols were entered into the software EATOS [5] in order to determine the waste per kilogram of product. The result is shown in Table S-1 and in Figure S-1. Software-assisted mass balance is a tool to identify issues that require optimization [6].

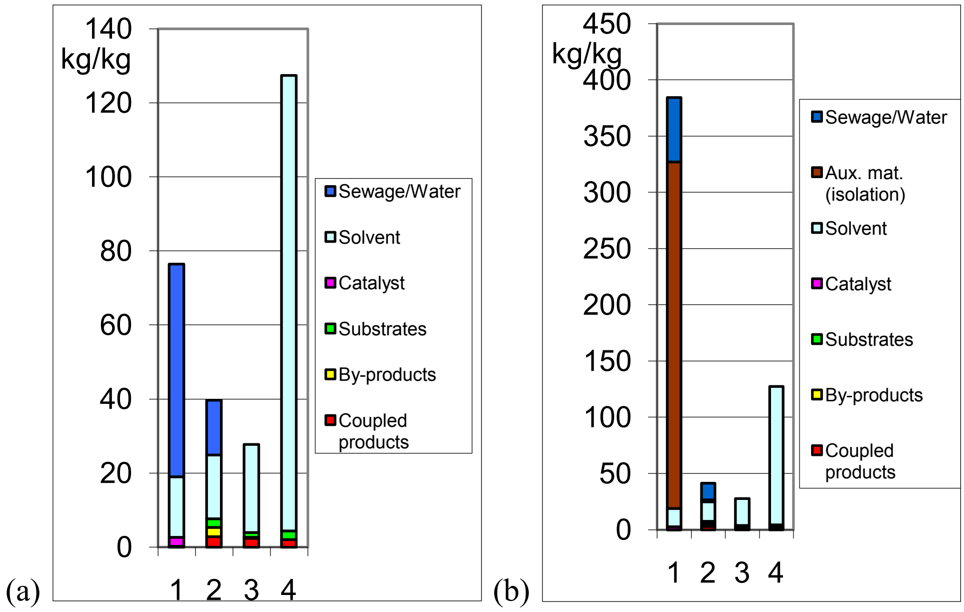

Figure S1: Environmental factor (E) of the deoxygenation methodologies shown in Schemes S-1 to S-4 numbered consecutively with 1 to 4. Whereas (a) only shows substances used during reaction, (b) also illustrates those being applied during work-up. As greater substance amounts are used altogether (reaction + isolation in (b)), the scaling is different.

Table S1: (i) Values shown in Figure S1. (ii) Substrate amounts that were used for the reaction. (iii) atom economies of the synthesis sequences shown in Scheme S1 to Scheme S4.

(i)

**Entry:**	**1**	**2**	**3**	**4**
(a)				
Coupled products	0.233	2.8862	2.4118	2.11
By-products	0.0514	2.4997	0.2284	0
Substrates	0	2.2933	1.3516	2.25
Catalyst	2.376	0	0	0
Solvent	16.4078	17.2271	23.7736	123
Sewage/Water	57.4502	14.8146	0	0
(b)				
Coupled products	0.233	2.8862	2.4118	2.11
By-products	0.0514	2.4997	0.2284	0
Substrates	0	2.2933	1.3516	2.25
Catalyst	2.376	0	0	0
Solvent	16.4078	17.2271	23.7736	123
Aux. mat. (isolation)	308.0334	1.8085	0	0
Sewage/Water	57.4502	14.8146	0	0

ii)

Entry:	1	2	3	4
Substrates	1.2843	8.6793	4.9919	6.13

iii)

Entry:	1	2	3	4
Atom economy	81	27	36	36

The hydriodic acid catalyzed reduction (entry 1 in Figure S1 (a) and Table S1) is, except for sewage production, the most mass-efficient reaction. The low amount of substrate demand (entry 1 in Table S1 (ii)), which is due to the high atom economy (Table S1 (iii)), results in the formation of only low amounts of coupled products or byproducts compared to entries 2 to 4 (Figure S1 (a) and Table S1 (i)). Additionally, some of the coupled products of entries 2 to 4 are supposed to be much more problematic with regard to waste treatment and recycling. In contrast, if hydriodic acid is not recycled it can easily be neutralized and disposed of in the sewage plant. The coupled product phosphoric acid will be easily flocculated in the treatment plant but will thereby contributing to the eutrophication problem. The catalytic use of iodine and the replacement of the stoichiometric reagent phosphorous, e.g., by an electrochemical method, are ways to improve the efficiency of the method. Another obvious optimization potential is the reduction of solvent and water during the work up.

References

1. Becker, H. G. O., et al., Organikum: Organisch-chemisches Grundpraktikum, 16 ed., VEB Deutscher Verlag der Wissenschaften, Berlin, **1986**. p 559.
2. Schmid, H.; Karrer, P., *Helv. Chim. Acta* **1949**, *32*, 1371-1378.
3. Dieguez, H. R.; Lopez, A.; Domingo, V.; Arteaga, J. F.; Dobado, J. A.; Herrador, M. M.; Quilez del Moral, J. F.; Barrero, A. F., *J. Am. Chem. Soc.* **2010**, *132*, *1*, 254-259.
4. Barton, D. H. R.; McCombie, S. W., *J. Chem. Soc., Perkin Trans. 1* **1975**, 1574-1585.
5. Eissen, M.; Metzger, J. O., *Chem. Eur. J.* **2002**, *8*, *16*, 3580-3585.
6. Eissen, M.; Weiß, M.; Brinkmann, T.; Steinigeweg, S., *Chem. Eng. Technol.* **2010**, *33*, *4*, 629-637.
